# Identification of ions in experimental electrostatic potential maps

**DOI:** 10.1107/S2052252518006292

**Published:** 2018-06-01

**Authors:** Jimin Wang, Zheng Liu, Joachim Frank, Peter B. Moore

**Affiliations:** a Department of Molecular Biophysics and Biochemistry, Yale University, New Haven, CT 06520-8114, USA; b Department of Biochemistry and Molecular Biophysics, Columbia University, New York, NY 10027, USA; c Department of Biological Sciences, Columbia University, New York, NY 10027, USA; d Department of Chemistry, Yale University, New Haven, CT 06520, USA

**Keywords:** cryo-EM, electron-scattering length, transition *B* factor, Mott equation, ribosome

## Abstract

Cryo-electron microscopy (cryo-EM) directly images the distribution of electrostatic potential (ESP) within macromolecules, and thus can provide much more information about atomic charge than X-ray crystallography. The electron-scattering length of an isolated ion is quite different from that of the corresponding neutral atom. The difference is very large at small scattering angles where the effects of electron distributions are largest, but becomes smaller at high scattering angles where nuclear charge determines outcomes. For this reason, in cryo-EM maps that have been solved at resolutions lower than ∼2.5 Å, peaks corresponding to anions will always be less prominent than those of cations, and may even be negative. Furthermore, if a map of this kind is smeared computationally after the fact, which reduces its effective resolution, anion peaks will diminish in size, cation peaks will grow and peaks that represent uncharged atoms will remain about the same. These effects can be used to determine the sign of the charges carried by the ions associated with a macromolecule and even estimate their magnitudes. The ESP value for a cation in a cation–anion pair is smaller than the value of the cation in isolation, but the ESP value for the anion in the ionic pair is greater than the value of the anion in isolation. The experimental range of ESP values for Mg^2+^ relative to that of the closest C1′ atom is found to be between 0.57 and 1.27.

## Abstract 2

Cryo-EM maps are sensitive to total nuclear and electron charges of a species whereas X-ray crystallography only visualizes electrons without a nuclear contribution. Cryo-EM can be used to identify charge and chemical identity.

## 1. Introduction   

Even though the electron-density (ED) distributions of macromolecules do respond to differences in atomic charges, their effects are so small that they are hard to detect in ED maps with resolutions worse than 1 Å. The electrostatic potential (ESP) maps of macromolecules provided by electron microscopy (EM) are much more sensitive to atomic charges and, paradoxically, the lower their resolutions, the larger the impact of charge. Here we show how the appearance of peaks corresponding to ions vary with the magnitude of the Δ*B* factor applied to the Fourier transforms of ESP maps after the fact, either to sharpen or blur them, and demonstrate that these effects can be used to determine the signs and the magnitudes of the charges carried by ions. Thus, species such as OH^−^, H_2_O, Na^+^ and Mg^2+^, which are almost indistinguishable in crystallographic ED maps because they contain the same number of electrons, should be easy to differentiate in ESP maps with modest resolutions.

The scattering length of an atom for electrons, *f* ^(e)^(*s*), is determined by the ESP distribution associated with it, to which both its nucleus and electrons contribute. By contrast, the scattering factor of an atom for X-rays, *f* ^(X)^(*s*), which is the ratio between its scattering and that of a single electron, is determined entirely by its ED distribution. While the two quantities are related [equation (1)], they are not interchangeable (Mott, 1930 ▸). As Mott demonstrated in 1930 ▸, the spherically averaged scattering length of an isolated atom (in Å) can be computed using the following expression:
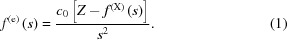
Here *Z* is the atom’s atomic number, *s* is the scattering vector defined as sin(θ)/λ, where θ is half the scattering angle, λ is the wavelength in Å and the constant *c*_0_ = *m*_0_ *e* ^2^/8π*h* ^2^ *∊*_0_ is 0.023934 Å^−1^ [see Peng (1999 ▸) for a historical review]. When the velocities of the electrons being scattered are appreciable compared with the speed of light, the mass assigned to them in the expression for *c*_0_ must be adjusted accordingly (Fujiwara, 1961 ▸).

The Mott equation [equation (1)] does not behave well at *s* = 0, *i.e.* when the scattering angle is zero, even if the atoms are uncharged, because the value it supplies for *f* ^(e)^(0) becomes indeterminate when *f* ^(X)^(0) = *Z*. It performs even worse for atoms carrying net charges because then [*Z* − *f* ^(X)^(0)] will equal the net charge and the value supplied for 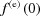 will be ± ∞. That said, the estimates for *f* ^(e)^(*s*) provided by the Mott equation for ions are accurate for *s* greater than 0.02 Å^−1^, provided that the X-ray scattering factors used, *f* ^(X)^(*s*), are those of the ion rather than those of the neutral species.

It is important to realise that the X-ray scattering factors of ions differ most from those of the corresponding neutral atoms at low scattering angles [*e.g.* pp. 477–499 in Wilson (1992) ▸], however, as stated earlier, the differences are not large for most ions, *e.g.* of the order of 10% for Na^+^ *versus* Na^0^. Charge effects are much bigger for electron-scattering lengths because of the subtraction required by the [*Z* − *f* ^(X)^(0)] term in the Mott equation. At a resolution of 10 Å, for example, the scattering length of an Na^+^ ion for electrons is about 2.6 times greater than that of a neutral Na atom, and the smaller the scattering angle, *i.e.* the lower the resolution, the greater the difference [*e.g.* pp. 226–243 in Wilson (1992 ▸)]. Therefore, it is not surprising that all of the (negatively charged) carboxylate residues in bacteriorhodopsin proved to be invisible in a cryo-EM map that was computed for that molecule using all the data available to a resolution of 3.0 Å, but were plainly evident in the cryo-EM map obtained from the same data set when all of the data of a resolution less than 7.0 Å were omitted (Kimura *et al.*, 1997 ▸).

These facts suggest that it might be possible to enhance the impact that net charge has on ESP maps by decreasing their effective resolutions after the fact. Here we show that this is in fact the case, and the effects that resolution manipulation has on ESP maps can be used to identify the peaks in those ESP maps that correspond to simple ions such as Na^+^, Mg^2+^ and Cl^−^, and to determine their charges.

## 2. Methods   

ESP maps were simulated in the space group *P*1 as described elsewhere (Wang & Moore, 2017 ▸), with *a* = *b* = *c* = 20 Å for the first simulation, *a* = *b* = *c* = 30 Å for the second and α = β = γ = 90° in both cases. Electron scattering lengths for neutral atoms and ions were taken from *International Tables for Crystallography* (Wilson, 1992 ▸) and also from data provided by Peng (Peng *et al.* 1998 ▸; Peng, 1999 ▸). There is very little numerical difference between the Mott equation and Peng’s parameterization of electron-scattering lengths for *s* < 2.0 Å^−1^ (*i.e.* 0.25 Å lattice resolution). The equivalent to equation (4) obtained using Peng’s scattering lengths is given elsewhere (Wang, 2018 ▸). The resolution of the simulated ESP maps examined was set at 2.50 Å by applying a *B* factor of +41.25 Å^2^ to the scattering lengths of all atoms. The value of *B* is derived from an empirical analysis of the X-ray crystallographic data sets deposited in the PDB (Wang, 2017 ▸). Explicit partial charges were included for all atoms using the values provided by Kollman and colleagues for nucleotides (Bayly *et al.*, 1993 ▸; Cornell *et al.*, 1995 ▸; Duan *et al.*, 2003 ▸), and the geometry and charge properties used for the [Mg(II)(H_2_O)_6_]^2+^ complex included in simulations were obtained from published sources (Pavlov *et al.*, 1998 ▸; Wang, 2017 ▸). Maps were analyzed and visualized using the graphics program *Coot* (Emsley & Cowtan, 2004 ▸). Figures were prepared using the program *PyMol* (Version 1.5.0.4, Schrödinger, LLC).

## 3. Results and discussion   

### 3.1. On the effects of disorder on the EM images of atoms   

For computational convenience, the X-ray scattering factors of atoms and ions are often represented as a sum of four Gaussians and a constant (Cromer & Mann, 1968 ▸; Fox *et al.*, 1989 ▸; Waasmaier & Kirfel, 1995 ▸),
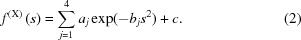
It follows that the electron-scattering lengths of atoms and ions can be written in the following form:
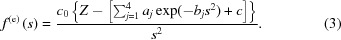
Disorder, such as that caused by the thermal motion of atoms, affects their appearance in the ED maps obtained by X-ray crystallography; it smears them out, and it has the same effect on the ESP maps obtained by cryo-EM. These smearing effects can be represented in reciprocal space by multiplying atomic scattering factors or scattering lengths, as the case may be, by Gaussians having the form exp(−*Bs* ^2^) or exp(−*BS* ^2^/4), where *S* = 2*s* = 2sin(θ)/λ. In crystallography, *B* is referred to as a temperature factor and its value can vary from one atom to the next in a molecule. The smearing effects caused by small errors in the image reconstruction process, by the point-spread function of the electron microscopes and the pixilation of detectors can also be represented the same way, though in this case all atoms are affected the same way.

Interestingly, the value of the ESP measured at the center of a peak representing an atom or ion in an ESP map is very sensitive to smearing because the ED component of its scattering length does not respond in the same way to smearing as its nuclear charge. That peak value 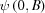 can be estimated by evaluating the following expression: 
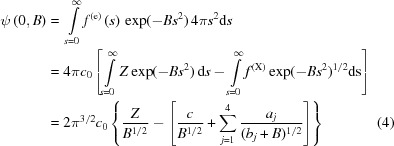
The integration called for converges only if *B* > 0, but because of the contributions made to *B* by the microscopes used to form images, in all of the circumstances of concern here this will always be the case. It is clear that the values of narrow peaks in ESP maps will fall more rapidly than those of broad peaks when maps are artificially smeared after the fact.

It may not be immediately obvious, but equation (4) predicts that if the *B* factor of an anion is large enough, the peak that represents it in an ESP map will be negative because the contribution made to that peak by its electrons will be greater than the contribution made by its nucleus. Thus, anions may be missed entirely if only positive features are taken into account when an ESP map is interpreted. If, on the other hand, the *B* factor of an anion is small, its nuclear term will dominate and the feature that represents it in an ESP map will be positive in the middle and will be surrounded by a negative halo.

The net charge carried by an ion affects not only the maximum value of the peak that represents it in an ESP map, ψ(*0*,*B*), but also the ESP values observed in its immediate neighborhood. That effect can be estimated using the standard expression for the electrostatic potential of point charges: Δ*q*/4π∊_o_ *r*, where Δ*q* is the net change of the ion, *r* is the distance from the nucleus of that ion to the point of interest and ∊_o_ is the dielectric constant. Polarization effects will tend to diminish the magnitude of these long-range contributions to the ESP maps, but their signs will always be that implied by the expression above. Interestingly, disorder has no effect on long-range potential contributions, provided the disorder is isotropic and the distance to the point of interest is large compared with the van der Waals radius of the ion.

### 3.2. The identification of Mg^2+^ cations in an experimental ESP map   

It follows from what has just been discussed that if an ESP map were deliberately smeared after the fact by multiplying all of its Fourier coefficients by a factor of exp(−Δ*Bs* ^2^) and then back-transforming them, one would find that the larger the value of Δ*B* used, the weaker the peaks that represent anions would become. By contrast, cation peaks would grow in significance as the smearing increased and the features representing uncharged atoms would remain about the same.

**Figure 1:**
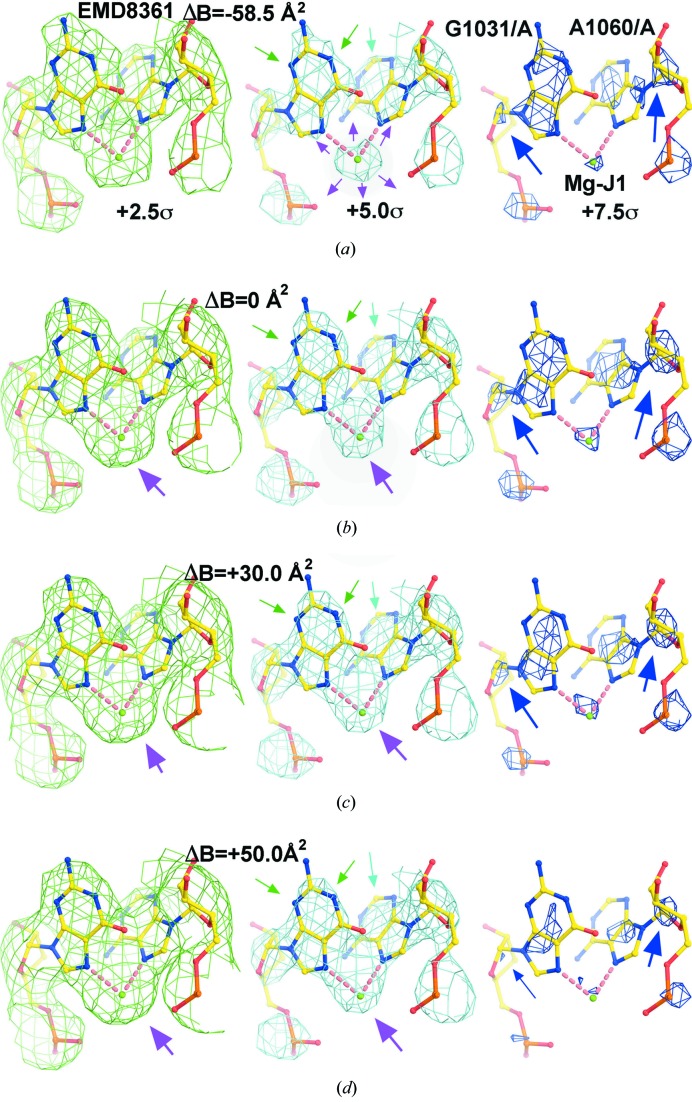
Experimental ESP map EMD-8361. (*a*) The ESP map with computational sharpening using Δ*B* = −58.5 Å^2^. (*b*) No sharpening, Δ*B* = 0Å. (*c*) Blurring, Δ*B* = +30 Å^2^. (*d*) Blurring, Δ*B* = +50 Å^2^. Maps are contoured at +2.5σ (left), +5.0σ (middle) and +7.5σ (right) for the Mg^2+^ site in question, known as the Mg-J1 site. Arrows highlight some notable features. Magenta arrows show that the volume for Mg^2+^ dramatically increases with increasing Δ*B* values, *i.e.* particularly at +5.0σ contouring level, whereas the volume for nucleobases remains unchanged, *i.e.* green arrows for N1 and N3 of G1031, and cyan arrows for C2 of A1060. Blue arrows indicate that the ESP value remains the highest on-atom ESP value on C1′ among all C atoms as well as among all the N and O atoms of the two nucleotides.

These inferences have been confirmed using the ESP map that was reported recently for the *Trypanosoma cruzi* ribosome (EMD-8361) at 2.5 Å resolution (Liu *et al.*, 2016 ▸, 2017 ▸). EMD-8361 is a sharpened version of an experimental map and the *B* factor used to sharpen it was −58.5 Å^2^. Such sharpening was reversed to regenerate the original, unmodified map, which therefore has a modifying Δ*B* factor of 0, and then two smeared versions of that map were computed, one having a Δ*B* factor of +30 Å^2^ and the other a Δ*B* factor of +50 Å^2^. Fig. 1 ▸ shows what the four maps look like in the immediate vicinity of the G1031/A1060 base pair where the peak that is assigned here as Mg-J1 is located (Mg-J1 is similar to a large number of other isolated ESP features in the EMD-8361 map that are unassigned in the coordinate file for PDB entry 5t5h). When the map is contoured at low levels, the only obvious effect of increasing Δ*B* is to make all of its features look more smeared than they otherwise would be. However, when these maps are contoured at higher levels, it becomes obvious that the larger the Δ*B* factor applied, the higher the maximum value of the peak assigned to Mg-J1 becomes, relative to other peaks (*i.e.* those for the nucleobases in the same map), and the lower the maximum value of the peaks that correspond to backbone phosphate groups. When these maps are contoured at the +5σ level, the volume occupied by the nucleobases is about the same in all of them, but the volume of the peak assigned to Mg-J1 increases greatly as Δ*B* increases (Fig. 1 ▸). There can be no doubt that the Mg-J1 peak in Fig. 1 ▸ represents a cation and the peaks assigned to the two neighboring phosphate groups are those of the anions. These observations are not unique to the Mg-J1 region of this map. The appearance of the peak that has been assigned as Mg-2011 ▸/A or Mg-J2, responds to changes in Δ*B* factor in exactly the same way (Fig. S1, see supporting information), and nearly all Mg sites previously assigned have the same property.

**Figure 2:**
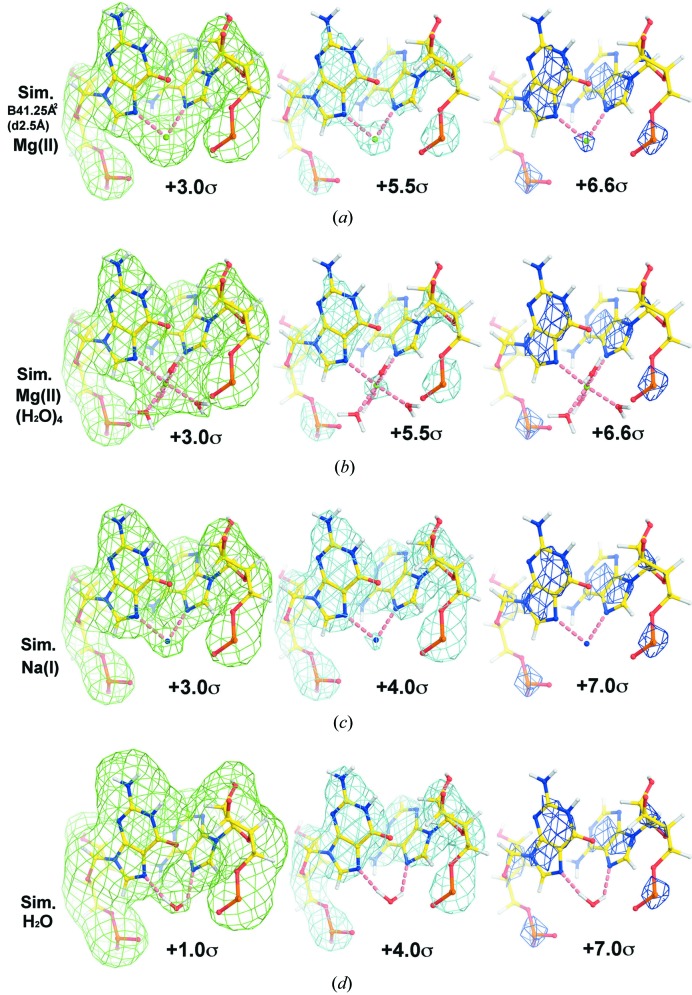
Simulated ESP maps for the Mg-J1 site using atomic models. (*a*)–(*d*) The model contains either Mg(II) or the [Mg(II)(H_2_O)_4_]^2+^ complex ion, Na(I) or H_2_O, and maps are contoured at three levels as indicated. The Fourier terms were limited to 2.5 Å and the mean atomic *B* factor of 41.25 Å^2^ was used.

The question of whether the cation peak identified as Mg-J1 should be assigned as an Mg^2+^ as opposed to an Na^+^, or an [Mg(II)(H_2_O)_4_]^2+^ complex ion (or even a H_2_O molecule), was addressed by comparing simulated ESP maps with the experimental map. For the purposes of these simulations, it was assumed that the *B* factors for all atoms and ions are the same and the *B* factor used was +41.25 Å^2^. This is the average *B* factor of all of the atoms in all of the macromolecular X-ray crystal structures deposited in the PDB that have resolutions close to the nominal resolution of EMD-8361 (∼2.50 Å) (Wang, 2017 ▸). As Fig. 2 ▸ shows, the simulated map obtained with an Mg^2+^ cation placed at the location in question is the one that most closely resembles the experimental Δ*B* = 0 map (Fig. 1 ▸). The same conclusion emerged when simulated maps having Δ*B* factors of +30 and +50 Å^2^ were compared with the corresponding experimental maps (data not shown).

The reader may have noticed that in the simulated ESP map for the dinucleotide model of the Mg-J1 site, the volume occupied by all positive features having magnitudes greater than +3.0σ is larger than the volume occupied by the same features in the experimental ESP map, even though the latter is contoured at a lower level (+2.5σ), but that the situation is reversed at higher contour levels (Figs. 1 ▸ *a* and 2 ▸ *a*). This non-linearity is not a result of errors in *B*-factor estimates (Fig. 1 ▸), but instead reflects the fact that the amplitudes of simulated and observed structure factors for the two maps cannot be scaled because the two maps have different density histograms. This is true of nearly all the high-resolution experimental ESP maps we have examined and its cause remains unknown.

In X-ray crystallography, the histograms of the ED maps of proteins are determined by the effective *B* factor of the diffraction data sets used to compute them (Nieh & Zhang, 1999 ▸). The *B* factor of a data set determines its resolution, and its value can be estimated by applying Wilson-plot methods to the data (Wang, 2017 ▸). Unfortunately, it is far less obvious what the meaning of the *B* factors is for many of the cryo-EM structures deposited in the PDB (Wlodawer *et al.*, 2017 ▸), and those uncertainties may make it hard to apply the ion identification methodology described in this study. It is also a concern that many of experimental ESP maps currently available were obtained by subjecting them to a variety of manipulations and/or filtering procedures that are poorly described and that users are likely to find correspondingly hard to understand, let alone to reverse.

## 4. The identification of Mg(II)(H_2_O)_*n*_ complex ions in experimental ESP maps   

In the simulated ESP map obtained for the Mg-J1 site, where there is an [Mg(II)(H_2_O)_4_]^2+^ complex ion bound, the four water molecules are clearly visible at low contour levels (Fig. 2 ▸ *b*). It appears that the positive contribution to the ESP made by charge of the Mg^2+^ beyond its own van der Waals radius increases the peak ESP value for the O atoms of those water molecules relative to those that have no cations nearby. Conversely, the negative contribution to the ESP in the region of the Mg^2+^ peak made by the partial negative charges of the four O atoms of these water molecules reduces the peak ESP value for the Mg^2+^ cation relative to that of an Mg(II) cation that has no bound water molecules (Figs. 2 ▸ *a* and 2*b*). Furthermore, the long-range negative contribution to the ESP in the region of water molecules by anions or atoms having negative partial charges (*i.e.* N7 atoms of purines, Figs. 2 ▸ *b* and 2*d*) decreases the peak ESP values for the O atoms of bound water molecules relative to those of isolated water molecules. The recognition that this kind of detail is evident in simulated ESP maps computed at moderate resolutions made us wonder if it might also be visible in experimental ESP maps. The analysis we carried out in order to identify the cation in the Mg-J2 site (Fig. S2), which we are about to describe, indicates that the answer to this question is affirmative.

It has long been known that Mg(II) prefers to form octahedral coordination complexes. Furthermore, if the number of RNA groups with which an RNA-bound Mg(II) interacts is large enough, it is possible to define the orientation of the axes of its coordination octahedron, and hence determine the locations where its other ligands, if any, should be found. The ligand orientation problem can be solved for both the Mg-J1 and the Mg-J2 sites, but inspection of a large number of the other Mg^2+^ sites in the EMD-8361 map (Liu *et al.*, 2016 ▸, 2017 ▸) showed that there is no way that this ESP map could be contoured as a whole that could be relied upon to reveal whether or not these cations have water ligands. Our alternative approach to review this matter was to examine plots displaying the variation in ESP along the octahedral axes of these ions in order to see if features could be identified at an appropriate distance from Mg(II) that could be reasonably assigned as water molecules.

**Figure 3:**
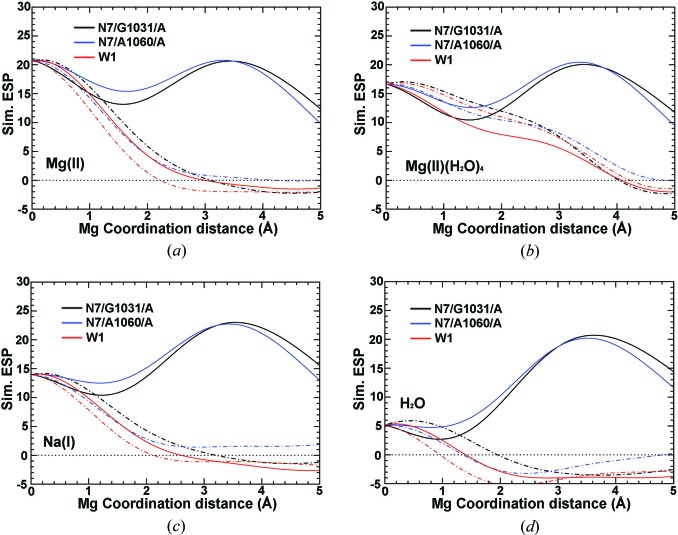
One-dimensional plots of simulated ESP maps centered at the Mg-J1 site. (*a*)–(*d*) The map contains either Mg(II) or Mg(II)(H_2_O)_4_, Na(I) or H_2_O. The Fourier terms were limited to 2.5 Å and the mean atomic *B* factor of 41.25 Å^2^ was used.

The above-mentioned one-dimensional plots that were obtained from the simulations shown in Fig. 2 ▸ reveal that there should be shoulder features at ∼2–3 Å from the Mg^2+^ center when H_2_O ligands are present, but not when they are absent (Fig. 3 ▸). A comparison of these simulated plots with those obtained for Mg-J1 shows that the vacant sites in the coordination sphere of the Mg(II) that are not occupied by RNA ligands are not occupied by ordered water molecules either. By contrast, similar analysis of the Mg(II) at the Mg-J2 site confirms the presence of two water molecules, W1 and W2, but no water molecule at the position that might be described as W3 (Fig. S3). It appears that W1 makes hydrogen bonds to two phosphate groups in the RNA backbone.

In the simulated ESP map, the shoulder feature for W1 is smaller than that of W2, but the opposite is true of the experimental data (Fig. S3). This difference could indicate that the partial charges carried by atoms in the phosphate groups of RNA backbones in this specific environment differ from those used in the simulation, which were taken from Kollman and colleagues (Bayly *et al.*, 1993 ▸; Cornell *et al.*, 1995 ▸; Duan *et al.*, 2003 ▸), particularly when phosphate–phosphate groups generate strong repulsive interactions.

To better understand why the W3 site of Mg-J2 is unoccupied, a third water molecule was built into the model *in silico* to see what would happen. It turns out that a H_2_O molecule cannot be placed at this location and avoid geometric clashes with the exocyclic N6-amine NH_2_ of A38/A and/or the C5H5 group of U37/A (Fig. S4). It appears that only a hydroxyl anion can be accommodated at this location. However, given that this Mg(II) interacts directly with the two phosphate groups and that there is a third phosphate nearby, this seems improbable.

## 5. Experimental considerations   

**Figure 4:**
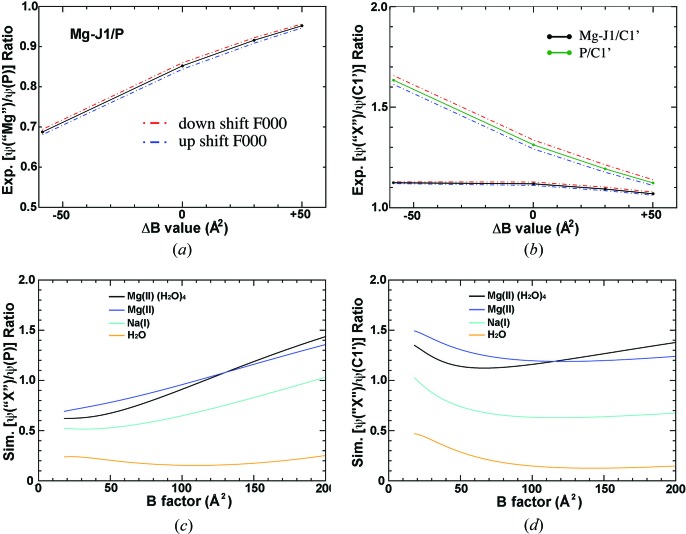
The experimental and theoretical ESP ratios at Mg-J1 site. (*a*) Experimental [ψ(‘Mg’)/ψ(P)] ratio (black) with a down shifted *F*(0,0,0) term (red) and an up shifted *F*(0,0,0) term (blue) added. (*b*) Experimental [ψ(‘*X*’)/ψ(C1′)] ratios for Mg-J1 and P. (*c*) Simulated ESP ratios in reference to the nearest P atom. (*d*) Simulated ESP ratios in reference to the nearest C1′ atom.

It is difficult to compare the ESP distributions produced by computer simulations with those obtained experimentally by cryo-EM because experimental ESP maps are not placed on the absolute voltage scale, and it is unclear what voltage the zero sigma contour level corresponds to. Uncertainties of this kind can be partly alleviated by using internal standards. The simulations performed suggest that the peak value of the potential at the C1′ atom closest to the ion of concern might be the best standard. The [ψ(*X*)/ψ(C1′)] ratio at the Mg-J1 site, for example, is almost completely independent of the Δ*B* value, where ψ(C1′) represents the ESP value on the nearest C1′ atom for the *X* atom (*X* = Mg^2+^, Na^+^ and H_2_O) (Fig. 4 ▸ *d*). Using Mg-J1 (ligands of nucleobases only) and Mg-J2 (multiple phosphate ligands) as representatives, it was found that [ψ(Mg^2+^)/ψ(C1′)] ratios are likely to vary between 0.57 and 1.27, the range of variation of [ψ(Na^+^)/ψ(C1′)] ratios lies between 0.36 and 0.54, and the corresponding range for [ψ(H_2_O)/ψ(C1′)] ratios is 0.25–0.29 (Figs. 4 ▸ and S5).

## 6. On the use of [ψ(*X*)/ψ(C1′)] ratios to identify Mg^2+^ peaks   

**Figure 5:**
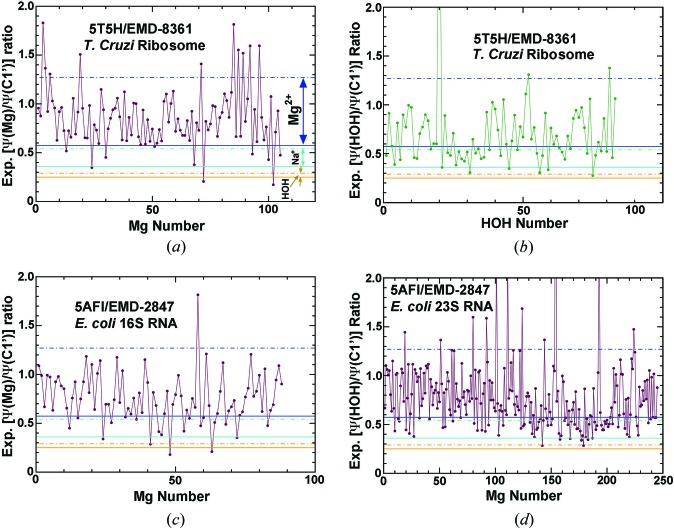
Application of the ESP ratios to two ribosome structures plotted as a function of the sequential order number of Mg^2+^ or H_2_O in the coordinate files. (*a*) Mg^2+^ in the 5t5h structure. (*b*) H_2_O in the 5t5h structure. (*c*) Mg^2+^ bound to the 16S rRNA in the 5af1 structure. (*d*) Mg^2+^ bound to the 26S rRNA in the 5af1 structure. The upper limits (dashed lines) of Mg^2+^ (blue), Na^+^ (cyan) and H_2_O (orange) are derived from the theoretical plot at Mg-J1 site assuming *B* = 41.25 Å^2^. The lower limits (solid lines) are from Mg-J2.

The cryo-EM structure of the large subunit of the *T. cruzi* ribosome cryo-EM structure (5t5h/EMD-8361) includes 105 Mg^2+^ cations and 83 ordered water molecules (Liu *et al.*, 2016 ▸, 2017 ▸); the 16S rRNA of *E. coli* ribosome has 88 Mg^2+^ and its 23S rRNA has 245 Mg^2+^ (Fischer *et al.*, 2015 ▸). When the experimental [ψ(*X*)/ψ(C1′)] ratios of the peaks assigned to Mg^2+^ were evaluated, it was found that >90% fall within the Mg^2+^ limits as expected. On the other hand, ∼75% of ESP peaks assigned as ordered water molecules in the file for structure 5t5h have ESP ratios that are within the Mg^2+^ limits (Fig. 5 ▸), which are much higher than expected for water molecules. The assignments of these ESP peaks need to be reconsidered.

## 7. Concluding remarks   

The visibility of charged species in the ED maps produced by X-ray crystallography is independent of the *B* factor, and at moderate resolutions, peaks corresponding to Mg^2+^, Na^+^ and H_2_O molecules are certain to be indistinguishable because they contain the same number of electrons. Thus, their assignment in ED maps must be based entirely on chemical context. As we have just shown, *B* factors have a much bigger effect on the visibility of these species in ESP maps. As *B* factors increase (*i.e.* effective resolution decreases), cation peaks increase in strength, and those of divalent cations grow faster than those of monovalent cations. Peaks that correspond to anions do the opposite; they get weaker. In fact, because of this effect, it can be difficult to visualize individual anions, or even H_2_O molecules in ESP maps with moderate resolutions, either simulated or measured. The observations described above indicate that these effects can be an advantage in determining the sign of the ion charge represented by peaks in ESP maps, and even their magnitude, perhaps through integration of the experimental charge-density maps in the space enclosing the species in question (Morse, 1932 ▸; Wang, 2017 ▸).

## Supplementary Material

Additional figures. DOI: 10.1107/S2052252518006292/kf5005sup1.pdf
