# Supplementary material for: Identification of ions in experimental electrostatic potential maps
Source: IUCrJ. 2018 Jun 1;5(Pt 4):375–81. doi: 10.1107/S2052252518006292 (PMC6038950; doi:10.1107/S2052252518006292)
Supplement: Supplementary file 1 [file m-05-00375-sup1.pdf]

IUCrJ

Volume 5 (2018)

Supporting information for article:

Identification of ions in experimental electrostatic potential maps

Jimin Wang, Zheng Liu, Joachim Frank and Peter B. Moore

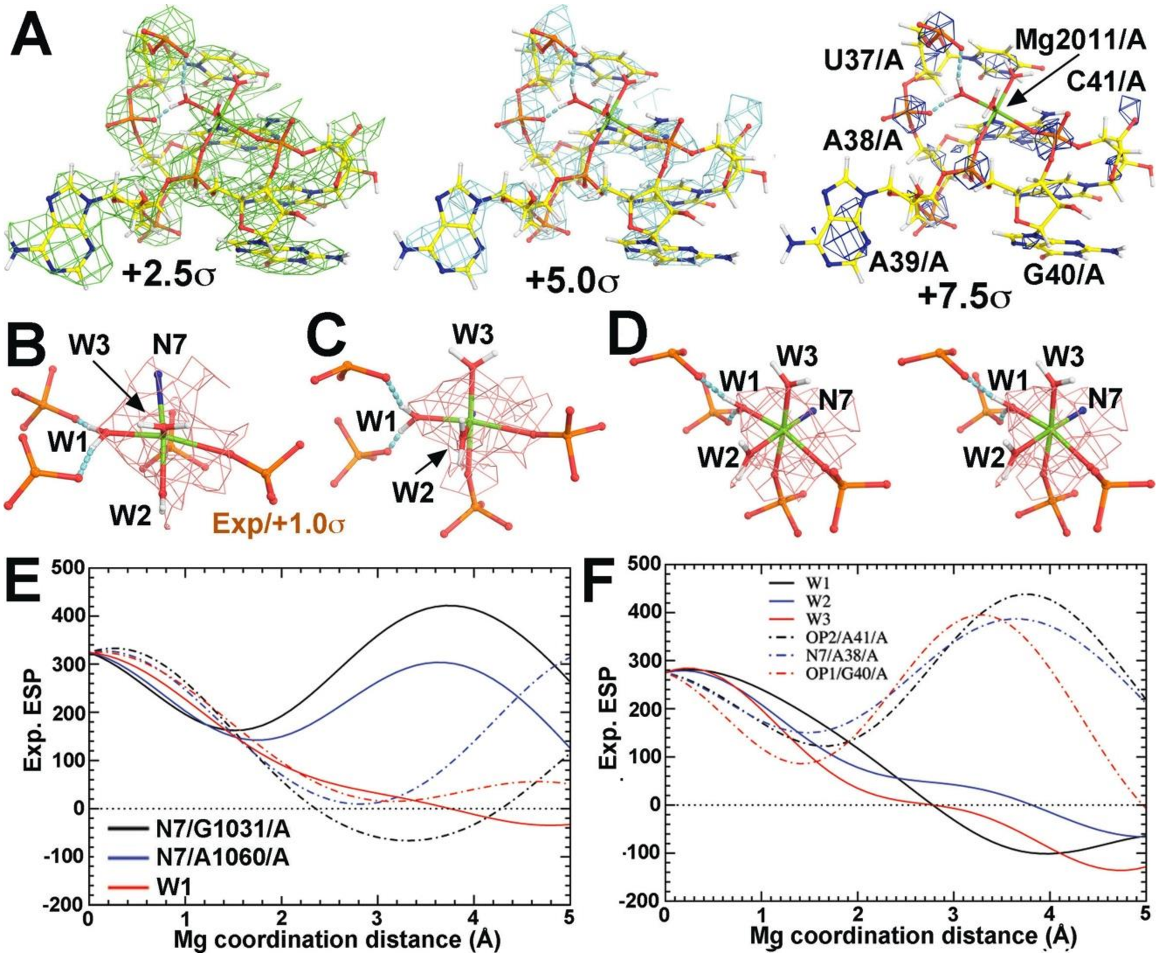

Figure S1. Experimental ESP maps (EMD-8361, Liu et al., 2016) for Mg(II) cations. (A) Mg binding site provided by OP2/A41/A, N7/A38/A, and OP1/G40/A, known as the Mg-A2011 site, superimposed on the ESP map contoured at +2.5σ (green, left), +5.0σ (cyan, middle), and +7.5σ (blue, right). (B, C) Two orthogonal close-up views of Mg-A2011 coordination, superimposed on the ESP map contoured at +1.0σ (salmon). (D) Stereodiagram of A2011 coordination. (E) Plot of experimental ESP centered at the Mg-J1 site as a function of coordination distance (Å) along the directions of eight ligands in three principle axes with three pairs of black, blue, and red (solid, and dashed) curves. (F) The experimental plot at the Mg-A2011 site. All experimental ESP functions are plotted using the same volume-scaling factor in this study.

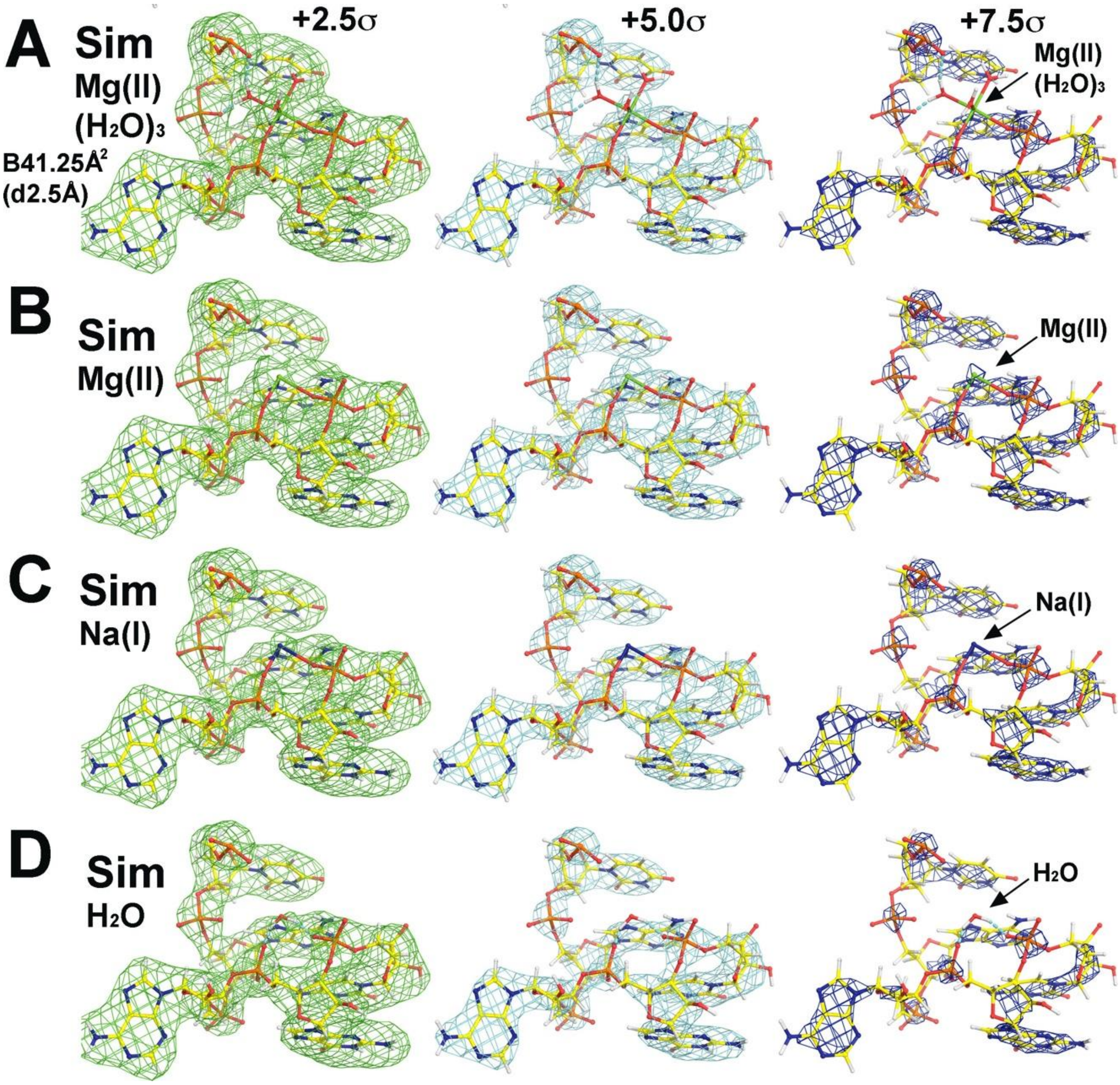

Figure S2. Simulated ESP for the Mg-A2011 site using Mg(II)(H_2O)$_3$ complex ion, Mg(II), Na(I), and H_2O (A-D) models contoured at three levels as indicated. The resolution was limited to 2.5 Å and atomic B-factor of 41.25 Å^2 was used.

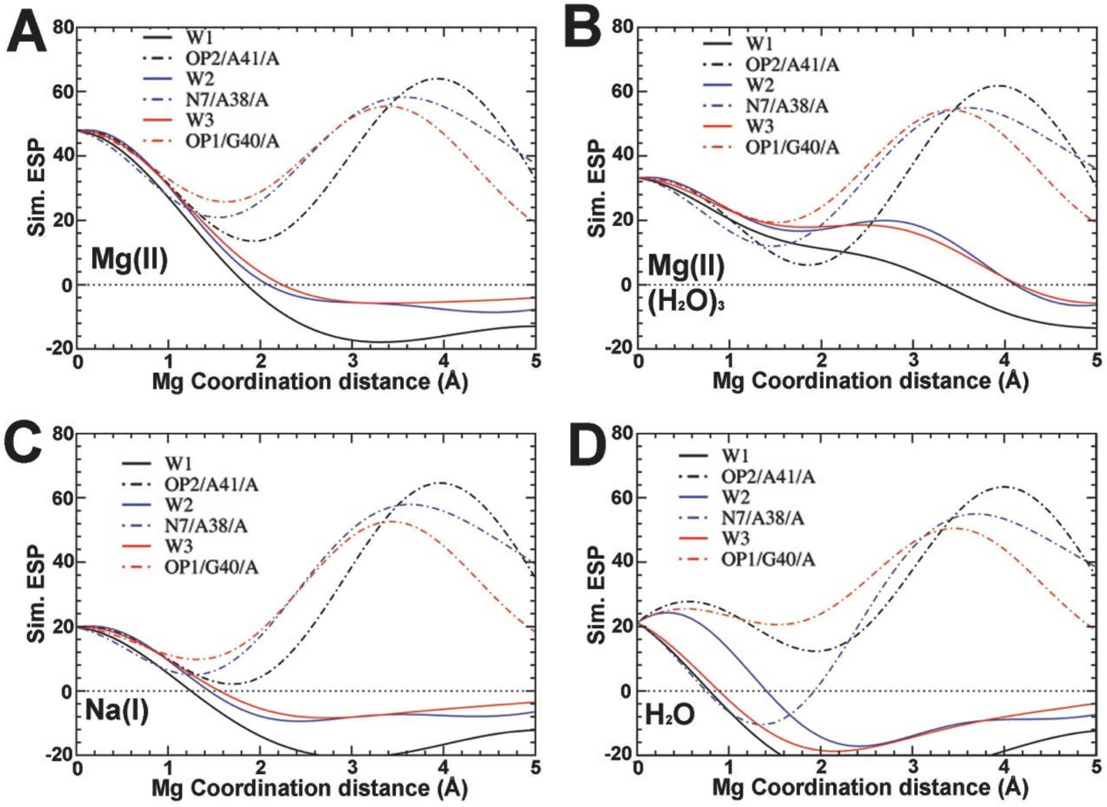

Figure S3. One dimensional plots of simulated ESP maps centered at Mg-A2011 site using Mg(II), Mg(II)(H_2O)$_3$, Na(I), and H2O (A-D). The resolution was limited to 2.5 Å and atomic B-factor of 41.25 Å^2 was used.

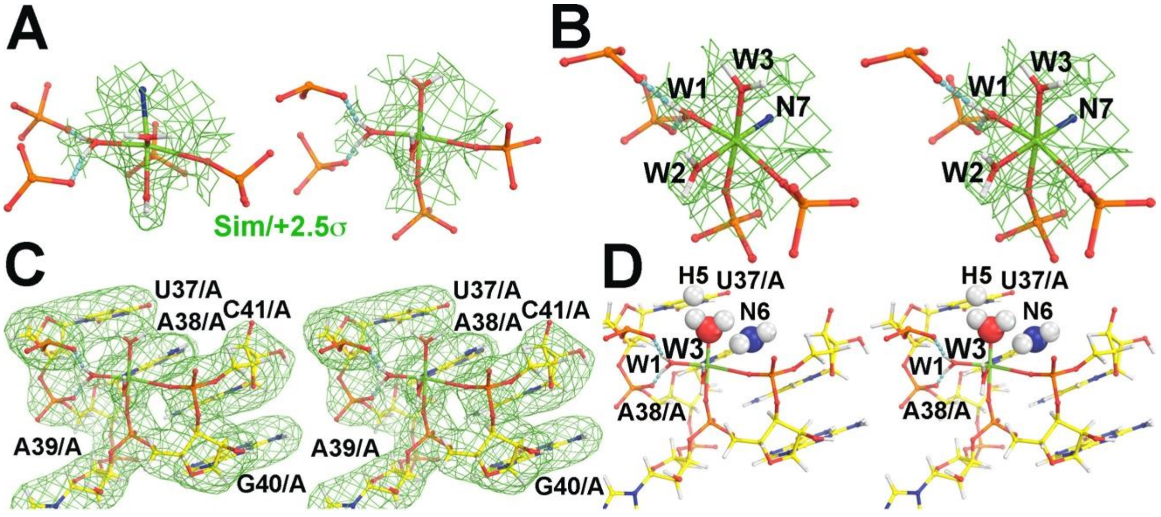

Figure S4. Simulated ESP map for Mg-A2011 site using Mg(II)(H_2O)$_3$ complex ion in close-up views contoured at +2.5σ (green). (A) Two orthogonal views. (B-D) Stereodiagams. (D) Predicted

van der Waals clashes between W3 and the exocyclic amine group of N6 of G1031/A, and the C5-H5 group of U37/A. The resolution was limited to 2.5 Å and atomic B-factor of 41.25 $Å^2$ was used.

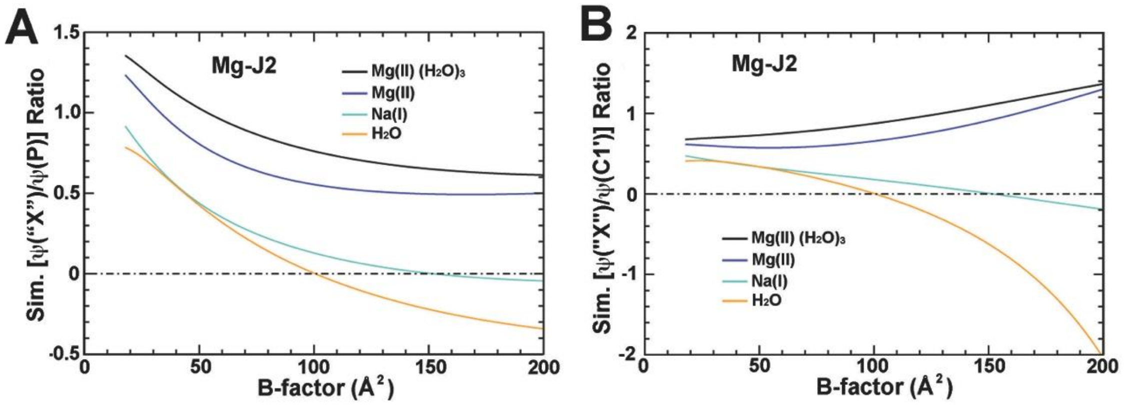

Figure S5. Simulated ESP ratios for species X at Mg-J2 sites.
